# Genome-Wide Identification of the *SnRK2* Gene Family and Its Response to Abiotic Stress in *Populus euphratica*

**DOI:** 10.3390/ijms262110750

**Published:** 2025-11-05

**Authors:** Hongyan Jin, Jing Li, Tongrui Song, Donghui Miao, Qi Ning, Xiao Zhang, Zhongshuai Gai, Zhijun Li, Peipei Jiao, Zhihua Wu

**Affiliations:** 1 Xinjiang Production and Construction Corps Key Laboratory of Protection and Utilization of Biological Resources in Tarim Basin, College of Life Science, Tarim University, Alar 843300, China; j2601803054@163.com (H.J.); jing926819729@163.com (J.L.); songtr20001209@163.com (T.S.); mdh1216g@163.com (D.M.); ningqi20000423@163.com (Q.N.); tlmdxzx@163.com (X.Z.); gaizhongshuaitea@163.com (Z.G.); lizhijun0202@126.com (Z.L.); 2 College of Life Sciences, Zhejiang Normal University, Jinhua 321004, China

**Keywords:** *Populus euphratica*, *SnRK2* gene family, drought stress, stomata

## Abstract

Improving plant water use efficiency (WUE) and drought tolerance by modulating stomatal activity constitutes a promising strategy for mitigating the impacts of water scarcity. *SnRK2*, a key component of the abscisic acid (ABA) signaling pathway, plays a critical role in modulating stomatal behavior under abiotic stress. However, the functional role of *SnRK2* in regulating stomatal movement to enhance WUE and drought tolerance in *Populus euphratica* remains to be characterized. In this study, 11 *PeSnRK2* genes were identified in the *P. euphratica* genome, each comprising 9–14 exons and exhibiting an uneven distribution across seven chromosomes. Subcellular localization predictions indicated that these proteins are predominantly localized in the Cytoplasm and Cytoskeleton. Phylogenetic analysis grouped the *PeSnRK2* genes into three distinct subfamilies, and conserved gene structures were observed within each clade. Analysis of cis-acting regulatory elements suggested that *PeSnRK2* genes were involved in hormonal signaling and stress response pathways. Further transcriptomic data also indicated substantial alterations in *PeSnRK2* expression due to polyethylene glycol (PEG) and abscisic acid (ABA) treatment. Finally, qRT-PCR and subcellular localization showed that *PeSnRK2.6* is highly induced by ABA and functions in both nucleus and cytoplasm. This first characterization in a desert woody species bridged gaps in *SnRK2* evolution and function.

## 1. Introduction

Global warming has intensified the severity of droughts worldwide, and as climate change progresses, droughts are projected to become more frequent and severe [1]. As sessile organisms, plants have evolved intricate mechanisms to cope with water deficit primarily through the modulation of signaling pathways [2,3]. Central to this adaptation is the dynamic regulation of stomatal aperture, which governs gas exchange and transpiration. Reduced stomatal conductance is a key water conservation strategy under limited water availability, and stomatal closure is a rapid and critical response that enhances drought tolerance by minimizing water loss [2,4,5].

Abscisic acid (ABA) is the primary regulator of stress-induced stomatal closure under drought conditions [6]. This phytohormone profoundly influences plant growth, development and responses to biotic and abiotic stressors [7]. During drought stress, *SnRK2s* that initiate the ABA signaling pathway are activated in plants, which comprises ABA receptors (PYR/PYL/RCAR), clade A protein phosphatase 2Cs (PP2Cs) and *SnRK2s* [8,9,10]. In the absence of ABA, PP2Cs repress SnRK2 kinase activity, thereby suppressing ABA signaling. Upon ABA binding, PYR/PYL/RCAR receptors bind to ABA and form complexes, relieving inhibition of *SnRK2s* and activating downstream signaling [11,12]. Water stress-induced ABA accumulation initiates a signaling cascade culminating in stomatal closure [13]. Specifically, ABA-bound PYR/PYL/RCAR receptors inhibit PP2C activity, permitting the autophosphorylation and activation of SnRK2 kinases. These activated *SnRK2s* subsequently phosphorylate downstream transcription factors governing stomatal closure and stress responses [14,15,16].

The Sucrose Non-Fermenting-1-Related protein Kinase (SnRK) family consists of evolutionarily conserved serine/threonine kinases ubiquitous in plants. Phylogenetically, this family is categorized into three subfamilies according to domain structure and function: SnRK1, SnRK2 and SnRK3 [17,18]. SnRKs can synergistically regulate stomatal development with auxin. The interaction between auxin and ABA signaling pathways suggests that SnRKs may act as integrators of these hormonal signals. *SnRK1/KIN10* and *SnRK2* interact with auxin signaling, modulating stomatal responses to abiotic stress through energy metabolism and stress response pathways [19]. Of these, SnRK2 is a plant-specific protein kinase family that plays a critical role in plant responses to stress. In *Arabidopsis thaliana*, 10 *SnRK2* genes (*AtSnRK2.1*–*2.10*) have been identified and classified into three groups according to their sensitivity to ABA. Group I (*AtSnRK2.1*, *2.4*, *2.5*, *2.9* and *2.10*) shows minimal or no response to ABA. Group II (*AtSnRK2.7* and *2.8*) exhibits weak ABA responsiveness. Group III (*AtSnRK2.2*, *2.3* and *2.6*) is strongly induced by ABA and is essential for ABA signaling and drought response [20,21,22,23,24,25,26]. Notably, *AtSnRK2.6* (*OST1*) plays a critical role in ABA-mediated stomatal closure. Loss-of-function mutants (*snrk2.6*) exhibit impaired stomatal regulation and excessive foliar water loss under drought stress [26,27]. The functional importance of *SnRK2s* has been demonstrated in various plant species apart from *Arabidopsis*, including dicots, such as *Fragaria vesca* [28], *Brassica rapa* [29], *Gossypium hirsutum* [30] and *Solanum tuberosum* [31], and monocots, such as *Oryza sativa* [18], *Zea mays* [32], *Triticum aestivum* [33] and *Saccharum officinarum* [34].

Advancements in computational power, coupled with the exponential expansion of biological datasets, have positioned bioinformatics as an indispensable discipline for addressing complex biological questions. In the present study, an integrated bioinformatics approach was employed, along with established software tools and curated online databases, for the systematic identification of *SnRK2* family members within the *Populus euphratica* genome. We further conducted a comprehensive characterization of these genes, including analyses of their physicochemical properties, chromosomal localization, conserved protein domains, *cis*-regulatory elements and phylogenetic relationships across species. These analyses provide critical insights into the potential functional roles of *PeSnRK2* kinases in abiotic stress responses.

*Populus euphratica* (commonly known as ‘desert poplar’) serves as a valuable model organism owing to its exceptional in vitro regeneration capacity, rapid clonal propagation and substantial ecological and economic relevance across Northern Hemisphere ecosystems [35]. As a halophytic relict species uniquely adapted to hyper-arid environments [36], *P. euphratica* provides an important system for investigating molecular mechanisms underlying abiotic stress tolerance, particularly drought and salinity resilience. Previous studies have demonstrated that overexpressing the ABA receptor *PYL4* upstream of the ABA signaling pathway or the downstream transcription factor *ABF3* can enhance poplar drought tolerance by regulating stomatal function [37,38]. While the biological functions of the SnRK2 kinase family and the key ABA signaling pathway gene *SnRK2.6* have been extensively studied across diverse plant taxa, their functional characteristics in woody perennial plants remain poorly characterized. We hypothesized that *PeSnRK2* genes, particularly those in SubGroup III, are up-regulated under drought and ABA, contributing to stress tolerance via nuclear signaling. This study presents a systematic characterization of the *SnRK2* gene family in *P. euphratica*, aiming to elucidate the regulatory functions of *PeSnRK2* genes in adaptive stress physiology. The findings not only provide new insights into the role of *PeSnRK2* in coping with abiotic stresses, but also provide a reference for the development and utilization of desert plant germplasm resources and for the restoration and reconstruction of vegetation, which is of great significance for ecological conservation.

## 2. Results

### 2.1. Identification and Characterization of the Physicochemical Properties of PeSnRK2 Family Members

Eleven *PeSnRK2* genes were identified in the *P. euphratica* genome, with properties detailed in Table 1. The physicochemical properties of the corresponding proteins were computationally characterized using ExPASy (https://web.expasy.org/protparam/; accessed on 16 November 2024). The analysis covered parameters such as polypeptide length, molecular weight, isoelectric point, instability index, aliphatic index, GRAVY, and predicted subcellular location. The protein lengths ranged from 338 amino acids (*PeuTF03G00739.1*) to 581 amino acids (*PeuTF05G01370.1*), with an average length of 381 amino acids. Molecular weights ranged from 38 kDa (*PeuTF03G00739.1*) to 65 kDa (*PeuTF05G01370.1*), with an average of approximately 43 kDa. The predicted pI values varied between 4.76 (*PeuTF09G01080.1*) and 9.57 (*PeuTF05G01370.1*), and most are acidic/weakly acidic, with only one being strongly alkaline. Acidic kinases may exhibit a greater propensity to phosphorylate basic regions on substrates (such as sequences near nuclear localization signals), whilst basic kinases may preferentially phosphorylate regions rich in acidic amino acids (e.g., glutamic acid, aspartic acid) on their substrate proteins. Instability index values ranged from 35.69 (*PeuTF05G01370.1*) to 50.77 (*PeuTF19G00050.1*), indicating potential instability under in vitro conditions. Unstable kinases permit cells to rapidly adjust their intracellular concentration through swift synthesis and degradation. This is characteristic of short-term signal responses. Stable kinases serve as enduring signaling scaffolds, participating in more fundamental, sustained cellular processes. All PeSnRK2 proteins exhibited negative GRAVY values, indicating their hydrophilic nature. Predictions of subcellular localization indicated that most *PeSnRK2s* are mainly found in the cytoskeleton and cytoplasm.

### 2.2. Analysis of Gene and Protein Structure of the PeSnRK2s

**Figure 1:**
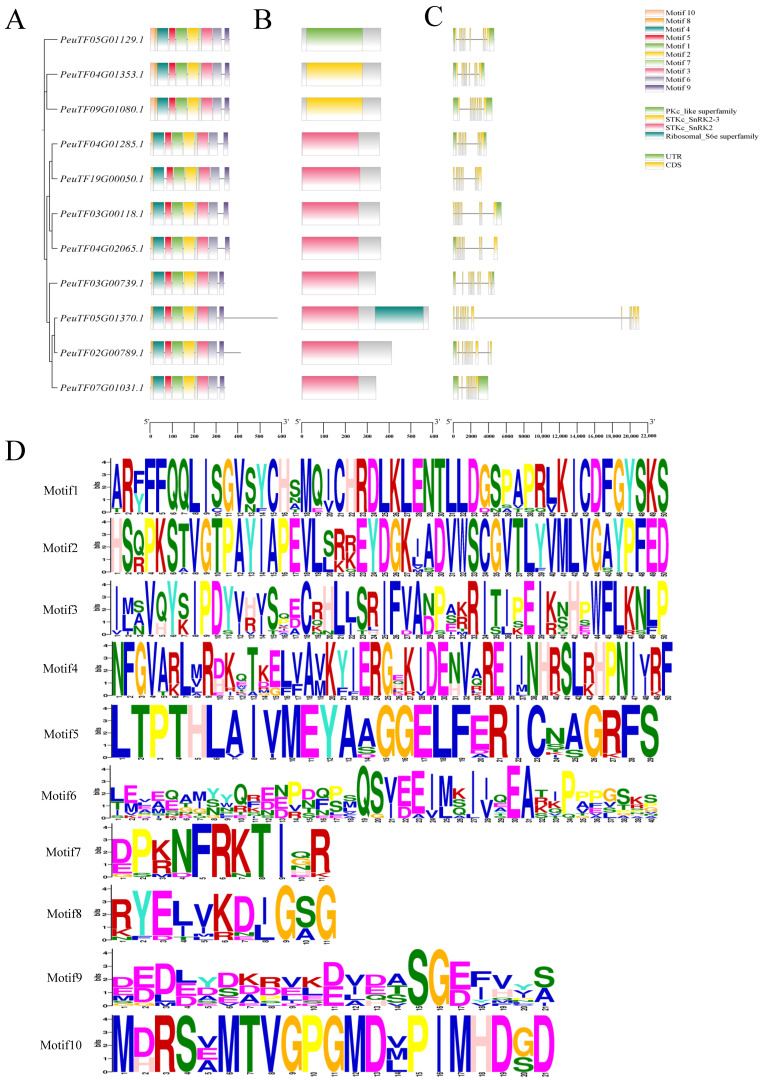
Analysis of gene structure and conserved structural domains of the *PeSnRK2* genes. (**A**) The distribution of conserved structural domains of *PeSnRK2* genes. (**B**) Pfam model of PeSnRK2s, 8 of the 11 *PeSnRK2* members have the STKc-SnRK2 domain model (green), 2 genes have the STKc-SnRK2-3 domain (yellow), 1 gene is characterized by the PKc-like-superfamily domain, and 1 gene is characterized by the ribosomal protein S6e domain. (**C**) The gene structures of *PeSnRK2* genes. (**D**) Conserved motif analysis of *PeSnRK2* proteins.

The gene structure of each *P. euphratica* SnRK2 family member was analyzed. Intron–exon grouping diagrams were constructed according to gene location information. Most members contained motifs 1–9, suggesting a high degree of conservation of these motifs. By contrast, motif 10 was present only in *PeuTF05G01129.1*, *PeuTF04G01353.1* and *PeuTF09G01080.1*, implying that motif 10 is less conserved within the family. Motif 10 is a sequence containing SH3 and RIO domains that can bind to target proteins, thereby altering their function by modifying enzyme activity, cellular localization, or interactions with other proteins (Figure 1A). Further domain analysis using the Pfam database confirmed that all PeSnRK2 proteins possess the canonical SnRK2 domain. Two members—*PeuTF04G01353.1* and *PeuTF09G01080.1*—also contained the SnRK2-3 subtype domain. In addition, the domain found in *PeuTF05G01129.1* was similar to the PKc superfamily, whereas *PeuTF05G01370.1* featured a ribosome-S6e domain (Figure 1B). The results showed that the number of exons in each member ranged from 9 to 14 (Figure 1C). The conserved protein domains of *P. euphratica SnRK2s* were identified using MEME v5.4.1 analysis, revealing 10 conserved motifs (motifs 1–10; Figure 1D). These findings are consistent with the domain architecture observed in *Arabidopsis SnRK2* family members, suggesting evolutionary conservation of core functional regions.

### 2.3. Prediction of Cis-Acting Elements in the Promoter Regions of PeSnRK2s

**Figure 2:**
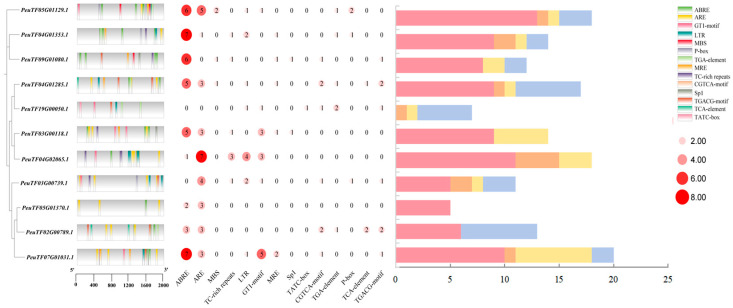
Cis-elements in the promoter region of *PeSnRK2s*. Color bars indicate the classification of cis-elements: pink bars represent biotic/abiotic stress; orange bars represent low-temperature responsiveness; yellow bars represent light-responsive elements; blue bars represent phytohormone response.

We conducted a systematic analysis of cis-regulatory elements within 2000 bp promoter regions to explore the functional diversification of PeSnRK2 genes. The forecasted cis-acting elements displayed varied distribution patterns among family members and were largely grouped into three main categories: hormone signaling, light responsiveness, and stress adaptation. Stress-related cis-acting elements were primarily associated with defense responses, drought tolerance and low-temperature adaptation. Hormone-responsive elements included salicylic acid-responsive elements (TCA-element), gibberellin-responsive elements (P-box and TATC-box), methyl jasmonate-responsive elements (CGTCA-motif and TGACG-motif), auxin-responsive elements (TGA-element) and ABA-responsive elements (ABRE), which are the key regulators of plant responses under abiotic stress conditions (Figure 2). Among the identified categories, hormone-responsive elements were the most prevalent, followed by light- and stress-responsive elements. Notably, ABREs demonstrated near-universal conservation, present in all PeSnRK2 promoters except PeuTF03G00739.1 and PeuTF19G00050.1. This evolutionary retention highlights ABA signaling as a central regulatory axis in *P. euphratica* stress adaptation mechanisms via SnRK2.

### 2.4. Collinearity Analysis of the SnRK2 Family in Multiple Species, PeSnRK2 Gene Duplication Types and Their Chromosomal Localization

Collinearity analysis can generally be divided into two categories. The first category is interspecific genomic collinearity analysis, which looks at the level of genome conservation and similarity between various species. The second type focuses on intraspecific collinearity, assessing homology among chromosomes within a single genome, and the distribution of repetitive sequences and multi-copy genes was assessed. Cross-species collinearity analysis provides crucial insights into the evolutionary development and expansion strategies of gene families. Such analysis provides a framework for elucidating the evolutionary dynamics of the *PeSnRK2* gene family and its potential role in drought adaptation, thereby identifying candidate genes for molecular breeding and genetic improvement. Hence, *SnRK2* gene family members were identified from the genomes of several related *Salicaceae* species. A total of 11, 14 and 10 *SnRK2* genes were identified in *Populus pruinosa*, *Populus deltoides* and *Salix sinopurpurea*, respectively.

**Figure 3:**
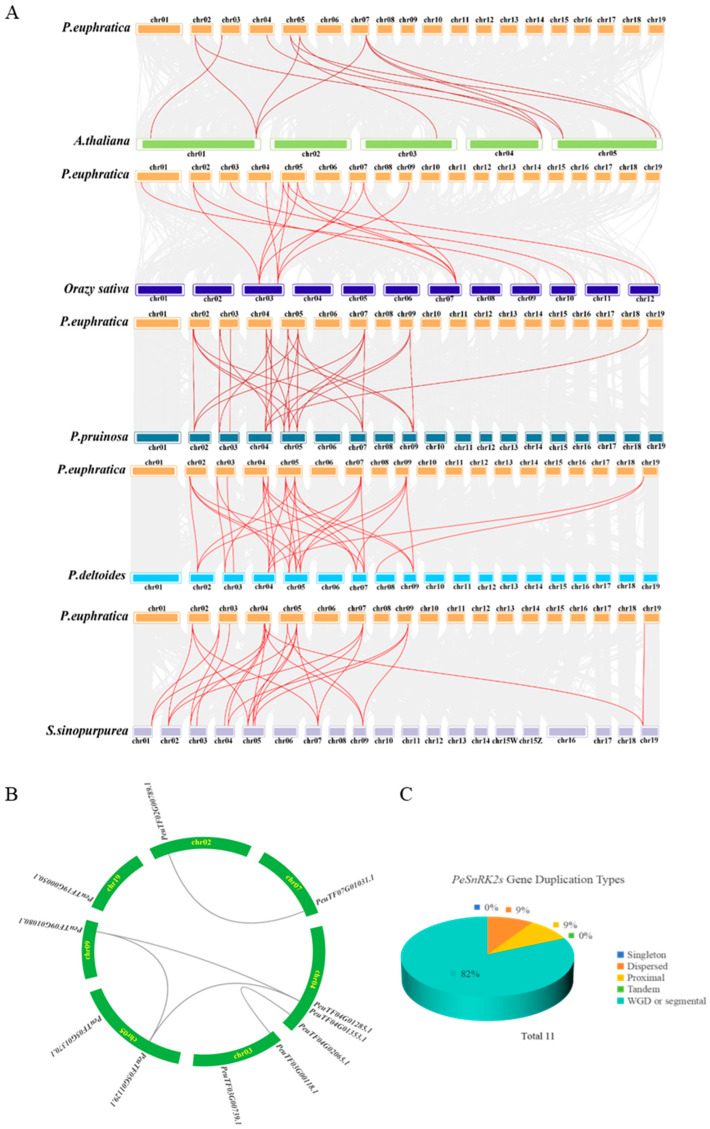
Collinearity analysis of *SnRK2s* from multiple species, *PeSnRK2* gene duplication types and chromosomal localization. (**A**) Collinearity analysis indicates that *P. europratica* shares collinearity with five other species (*P. pruinosa*, *P. deltoides*, *S. sinopurpurea*, *A. thaliana* and *O. sativa*). The gray lines in the background indicate collinearity groups within the *P. europratica* and other plant genomes, while the red lines highlight collinear *SnRK2* pairs. (**B**) Intraspecific collinearity analysis of *PeSnRK2s*. (**C**) Gene duplication types of *PeSnRK2s*.

To further investigate the evolutionary trajectory of the *PeSnRK2* gene family, collinearity analyses were conducted between *P. euphratica* and three other Salicaceae species (*P. pruinosa*, *P. deltoides* and *S. sinopurpurea*) and two model plant species: the dicot *A. thaliana* and monocot *O. sativa*. A total of 27, 31, 26, 12 and 15 collinear *SnRK2* gene pairs were identified between *P. euphratica* and *P. pruinosa*, *P. deltoides*, *S. sinopurpurea*, *A. thaliana* and *O. sativa*, respectively. These results demonstrate a higher degree of evolutionary conservation and orthology of *SnRK2* genes within the *Populus* genus than within the more distantly related species, such as *A. thaliana* and *O. sativa* (Figure 3A). Intraspecific collinearity analysis within *P. euphratica*, performed using the MCScanX function in TBtools, revealed five segmental duplication events involving seven of the 11 *PeSnRK2* genes: *PeuTF09G01080.1/PeuTF05G01129.1*, *PeuTF09G01080.1/PeuTF04G01353.1*, *PeuTF05G01129.1/PeuTF04G01353.1*, *PeuTF03G00118.1/PeuTF04G02065.1*, *PeuTF07G01031.1/PeuTF02G00789.1* and *PeuTF09G01080.1*/*PeuTF04G01129.1* (Figure 3B). All segmental duplication events occurred between different chromosomes but within three distinct subfamilies. These results suggest that multiple *PeSnRK2* genes arose during gene duplication, and segmental duplication events may have contributed to *SnRK2* gene expansion in *P. euphratica*. Gene duplication mode analysis further showed that whole-genome duplication or segmental duplication accounted for 82% of the *PeSnRK2* genes. By contrast, scattered duplication and proximal duplication each contributed 9%, while no tandem or singleton duplication events were observed (Figure 3C). The *PeSnRK2* fragment replication pattern may be associated with gene functional differentiation. The ratio of Ka/Ks is generally used to assess selective pressure and the evolutionary divergence of protein coding genes [39]. We estimated the Ka, Ks, and Ka/Ks ratio of homologous genes in *P. euphratica* (Table S1): their Ka/Ks ratios were all less than 1. These results suggested that the *PeSnRK2* gene family underwent purifying selection after duplication.

The number of *PeSnRK2* genes (11 members) is slightly greater than that of *AtSnRK2* (10) and *OsSnRK2* (10). This suggests that during the evolutionary history of *P. euphratica*, its *SnRK2* gene family may have undergone a minor species-specific expansion, potentially providing richer regulatory potential for the poplar’s adaptation to specific environments. The higher number of collinear pairs between *PeSnRK2s* and *OsSnRK2s* (15 pairs) compared to those with *AtSnRK2s* (12 pairs) indicates that poplar is evolutionarily closer to monocotyledons (rice). This suggests poplar may have inherited more conserved duplication units from a common ancestral gene or maintained a high degree of genomic structural conservation following divergence.

**Figure 4:**
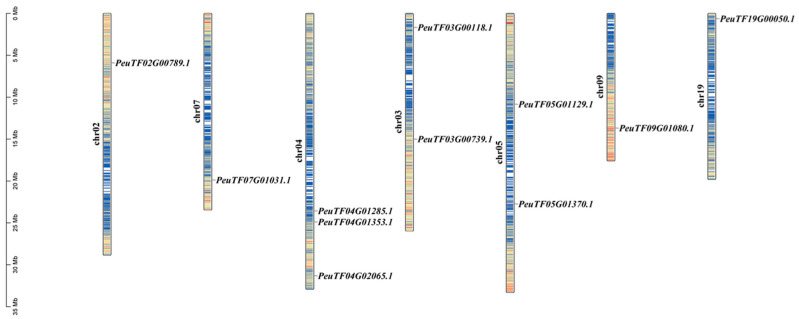
Analysis of chromosomal localization of *PeSnRK2s*. Blue, yellow and red bars represent low, medium and high gene densities on the chromosomes, respectively.

Chromosomal localization analysis of these 11 *PeSnRK2s* revealed that they are distributed across 7 of the 19 chromosomes of *P. euphratica* (Figure 4). Chromosome 4 contains the highest number of *PeSnRK2s* (three *PeSnRK2s*), followed by chromosomes 3 and 5 (two *PeSnRK2s*).

### 2.5. Systematic Evolutionary Analysis of the SnRK2 Gene Family in P. euphratica, P. pruinosa, A. thaliana and O. sativa

**Figure 5:**
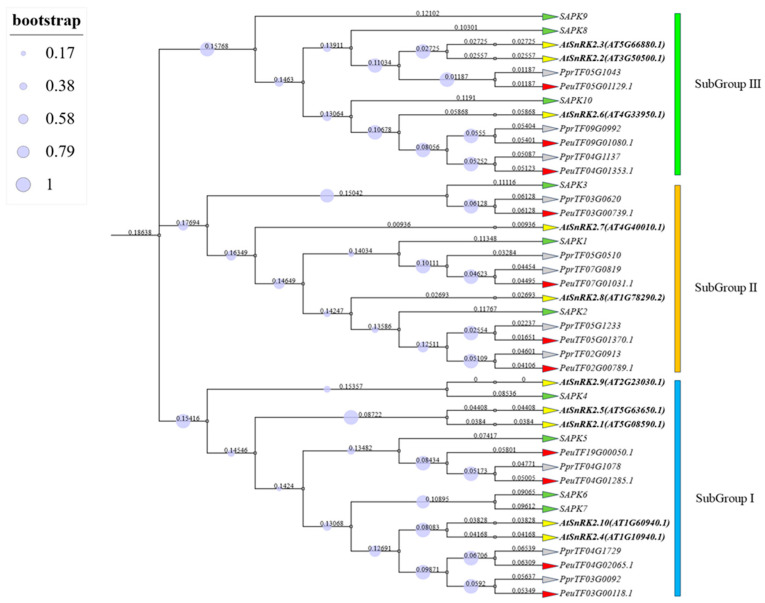
Dendrogram tree of *SnRK2* genes of *P. euphratica*, *P. pruinosa*, *A. thaliana*, and *O. sativa*. The neighbor-joining (NJ) tree was constructed using MEGA-X with 1000 bootstrap replicates to assess node reliability (values shown on branches). Evolutionary distances were calculated using the p-distance method, expressed as the number of amino acid substitutions per site. Bootstrap values <50 were disregarded. The gene tree of dendrogram was visualized using TBtools and the iTOL online website (https://itol.embl.de/, accessed on 5 March 2025). Gray triangles represent *P. pruinosa*, red triangles represent *P. euphratica*, yellow triangles represent *A. thaliana*, and green triangles represent *O. sativa*. Based on the topology of the gene tree, the *SnRK2* gene family was divided into three subgroups: I–III. The green band represents subgroup I, the orange band represents subgroup II, and the blue band represents subgroup III.

We constructed a gene tree using full-length protein sequences from the 11 *PeSnRK2* members of the woody dicotyledon *P. euphratica*, 10 *OsSnRK2* members from the herbaceous monocotyledon *O. sativa*, 10 *AtSnRK2* members from the herbaceous dicotyledon *A. thaliana* and 11 *PpSnRK2* members from the closely related woody dicotyledon *P. pruinosa* to investigate the evolutionary relationships among *PeSnRK2* genes. Based on the established subgroup classification from *Arabidopsis* and *Oryza*, the *PeSnRK2* members were assigned to three distinct subgroups: Subgroup I (*PeuTF03G00118.1*, *PeuTF04G02065.1*, *PeuTF04G01285.1* and *PeuTF19G00050.1*), Subgroup II (*PeuTF02G00789.1*, *PeuTF05G01370.1*, *PeuTF07G01031.1* and *PeuTF03G00739.1*), and Subgroup III *(PeuTF04G01353.1*, *PeuTF09G01080.1* and *PeuTF05G01129.1*; Figure 5). Comparative analysis revealed differential gene expansion within the *PeSnRK2* family relative to *A. thaliana*. Notably, two *PeSnRK2* genes (*PeuTF04G01353.1* and *PeuTF09G01080.1*) clustered with *AtSnRK2.6*, whereas another pair (*PeuTF05G01370.1* and *PeuTF02G00789.1*) grouped with *AtSnRK2.8*, suggesting the functional conservation in physiological and biochemical processes. Furthermore, phylogenetic proximity indicated that *P. euphratica* shares a closer evolutionary relationship with *P. pruinosa* than with *A. thaliana* or *O. sativa*.

### 2.6. Prediction of the Secondary Structure and Three-Dimensional Structure of the PeSnRK2 Protein

**Figure 6:**
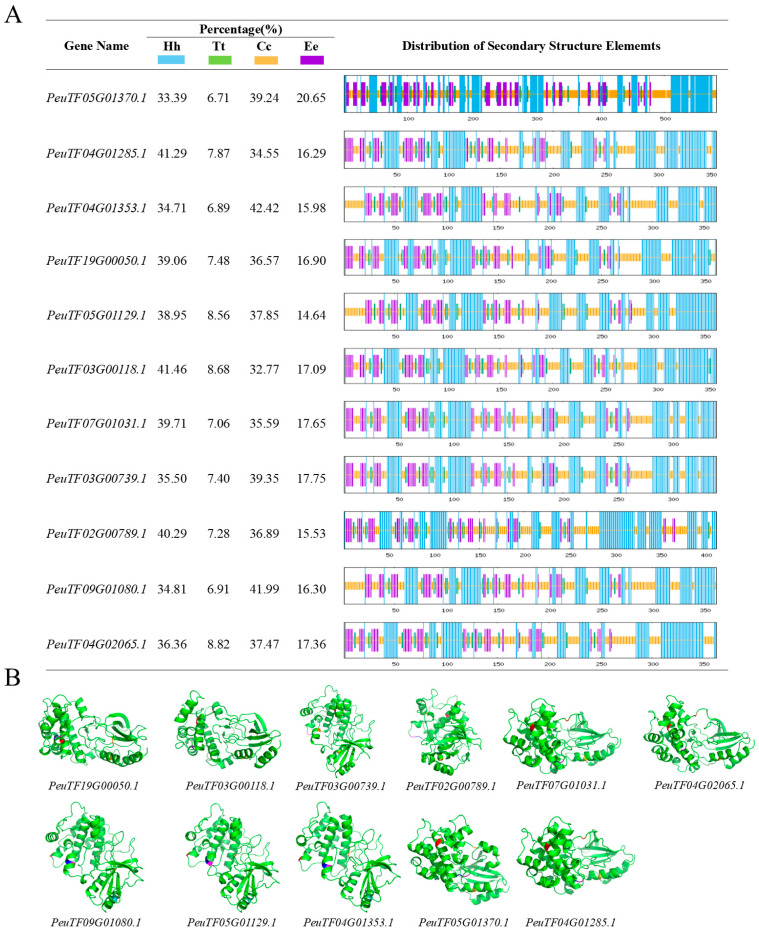
Secondary structure and 3D structure prediction of PeSnRK2 proteins. (**A**) Secondary structure prediction of PeSnRK2 proteins, Hh (alpha helix), Tt (beta turn), Cc (random coil), and Ee (extended strand). (**B**) Protein 3D structure prediction model of *PeSnRK2* gene families. Red denotes the DFG motif, yellow represents the VAIK motif, orange indicates the HRD motif, cyan signifies the ABA box, while purple and blue denote the phosphorylation sites S175 and S176 on the activation loop.

All PeSnRK2 proteins contain four secondary structures: α-helices, β-turns, random coils, and extended strands (Figure 6A). Random coils were the most abundant, followed by α-helices and extended strands. The predominance of α-helices and random coils suggests that these two structural features constitute the primary conformational framework of PeSnRK2 proteins. Random coils play a vital role in maintaining protein flexibility by facilitating conformational changes and enabling the connection between the more rigid α-helices and β-sheet structures. This flexibility is largely stabilized by hydrogen bonding interactions between backbone atoms or between backbone and side chains. α-Helices, which are stabilized through regular intra-chain hydrogen bonds, are critical for protein stability, often forming the core of functional domains and contributing to molecular interactions and protein folding. Three-dimensional structural modeling further revealed that all PeSnRK2 proteins exhibit highly similar tertiary conformations. Structural alignment showed that sequence identities exceeded 40% across all members, with the serine/threonine-protein kinase SRK2E serving as the homologous structural template. PeSnRK2 protein kinases all contain conserved core catalytic residues, including K50 (VAIK motif), D160 (HRD motif) and N191 (DFG motif), which collectively form the catalytic machinery for phosphate transfer. Furthermore, S175/S176 on the activation loop and the C-terminal D313 (ABA box) serve as crucial regulatory residues, governing the kinase’s conformational switch and interaction with the upstream inhibitor PP2C, respectively. Only subgroup III members harbor the ABA box, potentially correlating with their ABA-inducibility (Figure 6B).

### 2.7. Expression Profiles of PeSnRK2 Genes in Response to Abiotic Stresses

**Figure 7:**
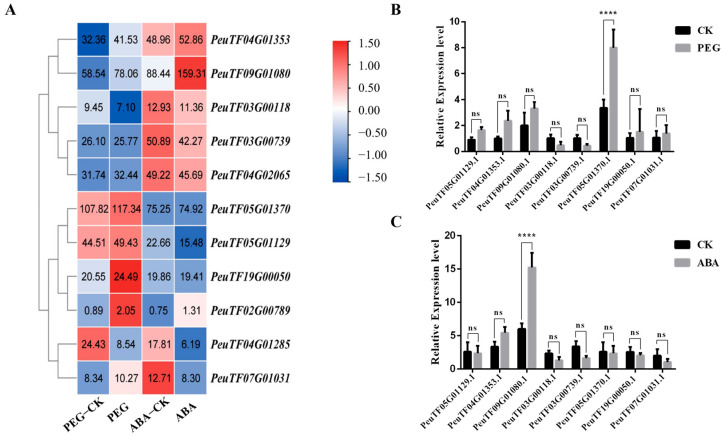
Expression patterns of *PeSnRK2s* under drought stress and ABA treatment. (**A**) Heat map of *PeSnRK2* expression patterns under drought stress (15% PEG 6000 treatment) and ABA treatment. The color scale indicating Z-score value; red indicates high levels, and blue indicates low levels of transcripts. (**B**,**C**) Validation of RNA-seq data by qRT-PCR, Statistical tests were performed by Sidak’s multiple comparisons test. The ‘****’ indicates significant differences of *p* < 0.0001; ‘ns’ indicates no significance.

Under PEG6000 treatment, five *PeSnRK2* genes, namely, *PeuTF04G02065.1*, *PeuTF05G01129.1*, *PeuTF07G01031.1*, *PeuTF19G00050.1* and *PeuTF05G01370.1*, were significantly up-regulated. By contrast, the expression levels of these genes were down-regulated in response to ABA. Three genes (*PeuTF03G00118.1*, *PeuTF03G00739.1* and *PeuTF04G01285.1*) exhibited reduced expression levels under both PEG-induced drought and ABA treatments (Figure 7A). Conversely, *PeuTF04G01353.1*, *PeuTF09G01080.1* and *PeuTF02G00789.1* were consistently up-regulated under both stress conditions. After normalizing the data, *PeuTF09G01080.1* demonstrated the most pronounced increase in transcript levels in response to ABA. Notably, this gene shares 89% sequence identity with *AtSnRK2.6*, a key regulator in ABA-mediated stress responses in *Arabidopsis*. Based on its strong inducibility and sequence homology, *PeuTF09G01080.1* was designated *PeSnRK2.6* and selected for subsequent functional characterization. Using qRT-PCR, we evaluated the expression levels of genes from eight members per branch of the phylogenetic tree. The results demonstrate that the expression patterns of all detected genes are broadly consistent with those observed in RNA-seq analyses (Figure 7B,C).

### 2.8. Subcellular Localization of PeSnRK2s

**Figure 8:**
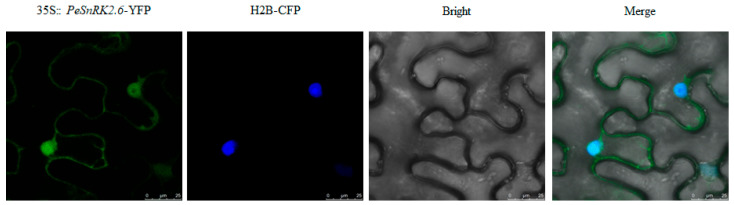
Nuclear localization of the 35S::*PeSnRK2.6*-YFP protein in tobacco leaf epidermal cells: fluorescence images of PeSnRK2.6 (35S::*PeSnRK2.6*-YFP), nuclear localization signals (H2B-CFP), and fusion images (35S::*PeSnRK2.6*-YFP/H2B-CFP). Scale bar = 25 µm.

Protein function is often influenced by their localization within cells. Mature proteins perform stable biological functions after localizing to specific subcellular organelles. The SnRK2 proteins are plant-specific serine/threonine (Ser/Thr) kinases that modulate stress-responsive gene expression by phosphorylating downstream substrates, thereby enabling adaptive responses in distinct tissues under adverse environmental conditions. To determine the subcellular localization of *PeSnRK2.6*, a recombination plasmid containing the *PeSnRK2.6* coding sequence fused to yellow fluorescent protein (YFP) under the control of the CaMV 35S promoter (35S::*PeSnRK2.6-YFP*) was introduced into *Nicotiana benthamiana* leaves via *Agrobacterium tumefaciens*-mediated transient transformation. Fluorescence signals were visualized through laser scanning confocal microscopy. The fusion protein *PeSnRK2.6*-YFP was observed to colocalize with the nuclear marker H2B, confirming its primary localization in the cell nucleus, with some cytoplasmic localization also noted. This finding was the same as in previous research [40] (Figure 8).

### 2.9. Predicting Protein–Protein Interactions of the PeSnRK2.6

**Figure 9:**
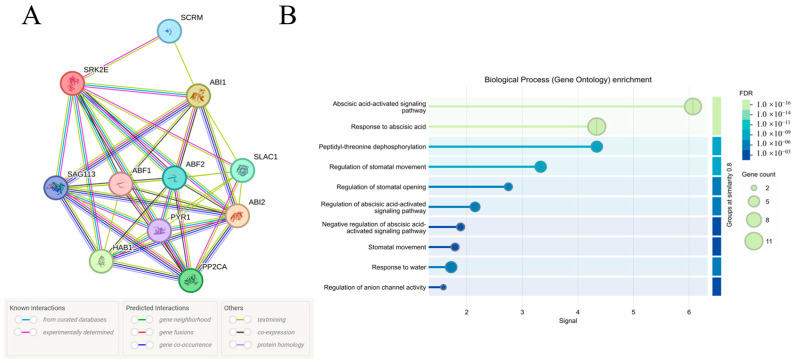
Functional network analysis of PeSnRK2.6 protein and associated signaling pathways. (**A**) Protein–protein interaction network of PeSnRK2.6. (**B**) Predicted signaling pathways.

To further elucidate the biological functions and regulatory networks of the *PeSnRK2.6* protein, a protein–protein interaction network was generated using the STRING database (http://string-db.org, accessed on 28 December 2024). Given the evolutionary conservation of SnRK2 kinases, the interaction network of PeSnRK2.6 was inferred according to its homologous protein, AtSnRK2.6 (also known as SnRK2E), in *A. thaliana*. The predicted interaction network revealed that SnRK2.6 interacts with several key regulators of ABA signaling, including *ABI1*, *ABI2*, *HAB1*, *PP2CA* and *SAG113*, all of which encode type 2C PP2Cs, the S-type anion channel *SLAC1*, the ABA receptor *PYR1*, ABA-responsive transcription factors *ABF1* and *ABF2* and the cold-response transcription factor *ICE1* (*SCRM*; Figure 9A). The functional annotation of this interaction indicates that *PeSnRK2.6* is likely involved in ABA-activated signaling pathways, ABA-mediated responses, peptidyl-threonine dephosphorylation and the regulation of stomatal movement (Figure 9B). These results suggest that *PeSnRK2.6* plays a central role in ABA-dependent stress response signaling networks in *P. euphratica*.

## 3. Discussion

### 3.1. Identification of 11 SnRK2 Family Members in P. euphratica

Protein kinase-mediated phosphorylation is a critical mechanism for mitigating ecological stress [41]. As central components of the serine-threonine kinase subfamily, SnRK2 kinases are indispensable to plant adaptation to environmental challenges, including drought [42,43]. These enzymes serve as key regulators within the ABA signaling transduction pathway [14], modulating gene expression and protein activity through target phosphorylation and coordinating osmotic stress responses and stomatal dynamics [44,45].

*SnRK2* genes have been isolated and identified in several species, such as 10 genes in *O. sativa* [18] and *A. thaliana* [46], 14 genes in *Z. mays* [32], 22 genes in tobacco [40]. In this study, 11 *SnRK2* genes were identified in both *P. euphratica* and *P. pruinosa,* 14 *SnRK2* genes in *P. deltoides,* 10 *SnRK2* genes in *S. sinopurpurea*. Bioinformatic analyses revealed conserved gene architectures, aligning with established structure–function relationships observed in plant kinase subfamilies. Previous research has indicated that *SnRK2* genes in most higher plants consist of nine exons, with some exceptions such as *ZmSnRK2.5* (one exon) [32], *OsSAPK5* (three exons) [47], and *AtSnRK2.8* (five exons) [48]. Typically, plant *SnRK2* genes contain eight introns, reflecting a high degree of structural conservation across species. Notably, the majority of *PeSnRK2* genes possess nine exons and eight introns, with the exception of two members of subgroup II (*PeuTF02G00789.1* and *PeuTF05G01370.1*) (Figure 2), consistent with the predominant exon number reported in *Nicotiana tabacum SnRK2* homologues [40]. This structural conservation across diverse higher plant taxa provides an evolutionary basis for future functional characterization of *PeSnRK2* kinases.

### 3.2. PeSnRK2 Family Members Respond to Abiotic Stress

The variation in gene expression levels is largely governed by the composition of cis-regulatory elements within its promoter region, which provide the crucial molecular foundations for responding to environmental stimuli. Promoter cis-acting element analysis of *PeSnRK2* genes elucidates their potential regulatory responses to environmental stimuli. In line with established mechanisms, ABA-responsive gene expression typically requires multiple ABREs or a single ABRE in conjunction with auxiliary motifs such as DRE/CRT [49]. In this study, ABRE motifs were identified in 9 of the 11 *PeSnRK2* promoters (Figure 2), consistent with observations in *Capsicum* [50], *Brassica napus* [51] and *Ammopiptanthus nanus* [52]. Subgroup III members (*PeuTF05G01129.1*, *PeuTF04G01353.1* and *PeuTF09G01080.1*) possess a relatively high number of AREB cis-acting elements and share motif10, suggesting they are strongly linked to ABA response. Additionally, the presence of low-temperature responsive elements within these promoters’ mirror patterns observed in *Liriodendron chinense SnRK2* genes [53], suggesting a conserved regulatory framework. These findings collectively imply functional roles for *PeSnRK2* genes in both ABA-mediated signaling and cold stress adaptation.

Genome-wide studies on plant responses to abiotic stresses are becoming increasingly thorough and systematic. *SnRK2* gene expression is widely induced by diverse abiotic stresses across plant species. In rice, *SnRK2* family members are regulated in an organ-specific manner by osmotic stress, salinity and ABA; *SAPK3*, *SAPK8*, and *SAPK9* were up-regulated by salt and drought, and *SAPK6* was transcriptionally activated by cold stress [18,46]. Similarly, in wheat, overexpression of *TaSnRK2.4* significantly increased the resistance of transgenic plants to salt, drought and freezing [54], while maize *SnRK2s* respond to both NaCl and ABA stimuli [32]. Overexpression of *MYB* transcription factors downstream of the *SnRK2* can enhance plant resilience against multiple abiotic stresses [55]. Based on these findings, we induced the expression of candidate genes in poplar trees under different treatment conditions involving PEG and ABA. In this study, two *PeSnRK2* genes were strongly induced by PEG and ABA treatments. *PeuTF05G01370.1* was strongly induced by PEG, while *PeuTF09G01080.1* was strongly induced by ABA. We further identified LTRs alongside multiple ARE and MRE elements within 7 of these 11 *PeSnRK2* genes. These elements interact synergistically with numerous cis-acting regulatory elements associated with abiotic stress responses, including ABRE and MBS (Figure 2). These key characteristics suggest that *PeSnRK2s* may play a pivotal role in transcriptional reprogramming events during the plant system’s response to diverse environmental stimuli, thereby ensuring the natural developmental growth process of poplar. However, the responsiveness of *PeSnRK2* genes to salt and low-temperature stress remains to be experimentally validated.

### 3.3. PeSnRK2.6 Enhances Plant Drought Tolerance by Regulating Stomatal Movement

Evolutionary analysis of *SnRK2* proteins in *P. euphratica* and *A. thaliana* enables inference of *PeSnRK2* expression patterns and regulatory functions based on phylogenetic relationships with well-characterized *AtSnRK2* genes. For example, *AtSnRK2.6* is predominantly expressed in guard cells and plays a central role in regulating stomatal aperture in response to abiotic stress [27]. In contrast, the closely related Group III members *AtSnRK2.2* and *AtSnRK2.3*, although phylogenetically proximate to *AtSnRK2.6*, exhibit broader tissue expression profiles and function primarily in ABA-mediated responses during key developmental stages, including seed germination, dormancy and early seedling growth [11]. Studies in *Populus tremula* have demonstrated that *Ptre-SnRK2.6a* and *Ptre-SnRK2.6b*, despite exhibiting high peptide sequence similarity, appear to exert distinct biological functions [56].

Phylogenetic analysis reveals that *PeSnRK2.6* shares 89% sequence identity with *Arabidopsis OST1/SnRK2.6/SnRK2E*, the primary regulator of stress-responsive stomatal movement [57]. This kinase plays a pivotal role in enhancing drought tolerance through ABA-mediated stomatal closure, a mechanism conserved across species including *A. thaliana*, *Camellia sinensis* [58], and *Zea mays* [59]. In *P. euphratica*, *PeSnRK2.6* expression is specifically induced by PEG-simulated drought stress and ABA treatment (Figure 6), suggesting its involvement in early drought perception and signal transduction. As a core component of the ABA signaling pathway, *PeSnRK2.6* likely operates through upstream PYR/PYL/RCAR receptors and downstream ABF transcription factors and regulates stress-responsive gene expression. We propose that *PeSnRK2.6* enhances drought resistance by phosphorylating key stomatal regulators, such as ion channels or transcriptional effectors, thereby promoting stomatal closure. While homology suggests roles, direct knockouts are needed to confirm. This study focuses on bioinformatics analysis and lacks functional validation through mutant or overexpressing plants; subsequent work will incorporate CRISPR editing technology or gene overexpression in poplar. Previous research indicated that overexpressing *AtSnRK2.8* in poplar may enhance abiotic stress tolerance via stomatal regulation [60]. This provides a reference for testing tolerance by overexpressing *PeSnRK2.6* in drought-sensitive poplar varieties. Given this functional conservation, *PeSnRK2.6* represents a promising candidate for genetic enhancement of drought tolerance in both woody perennials and crop species.

## 4. Materials and Methods

### 4.1. Identification and Physical and Chemical Property Analysis of Members of the PeSnRK2 Family

*PeSnRK2* genes were identified from the genomic dataset of *P. euphratica* [61]. Initial identification involved aligning the *P. euphratica* genome with protein sequences of the 10 *A. thaliana* SnRK2 family members via BLASTp website (e-value ≤ 1.0 × 10^−10^) (https://blast.ncbi.nlm.nih.gov/Blast.cgi, accessed on 15 October 2024). To detect potential SnRK2 proteins, HMMER searches were performed using the HMM profile of the SnRK2 domain (Pfam: PF00069) (http://pfam.xfam.org/search, accessed on 17 November 2024). Candidate sequences were further validated by screening for complete conserved domains using Pfam batch sequence search (http://pfam.xfam.org, accessed on 19 November 2024) and NCBI’s Conserved Domain Database (CDD) batch search (https://www.ncbi.nlm.nih.gov/cdd, accessed on 19 November 2024) and confirmed using SMART (http://smart.embl-heidelberg.de, accessed on 19 November 2024). The physiochemical properties of the identified PeSnRK2 proteins, including theoretical isoelectric point and molecular weight, were predicted using the ExPASy ProtParam tool (https://www.expasy.org, accessed on 24 November 2024). Subcellular localization predictions were conducted using WOLF PSORT (https://www.genscript.com/, accessed on 24 November 2024).

### 4.2. Genetic Structure and Conserved Domain Analysis of Members of the PeSnRK2 Family

Conserved motifs in the *PeSnRK2* proteins were identified using the web-based software MEME v5.4.1 (http://meme-suite.org, accessed on 28 November 2024). The motif discovery parameters were set with a minimum and maximum motif width of 10 and 150 amino acids, respectively, and the maximum number of motifs limited to 10. All other settings were kept at default values. Gene structure and motif distribution diagrams were visualized and analyzed using TBtools (Version 2.119).

### 4.3. Analysis of Cis-Acting Elements of PeSnRK2s

To investigate the regulatory elements and potential functional roles of *PeSnRK2* genes, we retrieved and analyzed 2000 bp upstream sequences from the translation start site of each *PeSnRK2* member, using the Plant CARE database (https://bioinformatics.psb.ugent.be/webtools/plantcare/html, accessed on 6 December 2024). The predicted cis-acting elements were subsequently visualized using TBtools software (Version 2.119).

### 4.4. Multispecies Collinearity, Gene Duplication Types and Chromosome Localization of PeSnRK2s

*SnRK2* family members from three additional *Salicaceae* species, namely, *P. pruinosa* (NCBI BioProject accession PRJNA863418), *P. deltoides* (WV94_445) [62] and *S. sinopurpurea* [63], were obtained using publicly available genome assemblies.

To investigate the evolutionary conservation and genomic synteny of *PeSnRK2* genes, homologous gene pairs between *P. euphratica* and five other species, namely, *P. pruinosa*, *P. deltoides*, *S. sinopurpurea*, *A. thaliana* and *O. sativa*, were identified through BLASTP alignment. Conserved genomic intervals between *P. euphratica* and these species were subsequently detected using TBtools (Version 2.119) and visualized. To further explore the evolutionary dynamics of the *PeSnRK2* gene family, we employed MCScanX (https://hmegasoftware.net; accessed on 14 December 2024) and analyzed whole-genome duplication events in *P. euphratica*. This analysis enabled classification of duplication types (e.g., segmental, tandem) and characterization of their distribution patterns within the genome. These findings provide insight into the expansion and evolutionary history of the *SnRK2* gene family in *P. euphratica*. TBtools software calculates the Ka and Ks values of duplicated genes.

Chromosomal localization of *PeSnRK2* genes in *P. euphratica* was determined using TBtools software (Version 2.119). Chromosome length information was obtained via the ‘Fasta Stats’ function, while gene IDs and positional data were extracted from the genome annotation (GFF3 file) using the ‘GFF3 Gene Position Parse’ and ‘Text Block Extract and Filter‘ tools. Gene density information was retrieved using the ‘Gene Density Profile’ module. Finally, the chromosomal distribution of *PeSnRK2* genes was visualized using the ‘Gene Location Visualize’ function, providing an overview of their physical positions across *P. euphratica* chromosomes.

### 4.5. Phylogenetic Analysis of PeSnRK2s

To investigate the evolutionary relationships among *SnRK2* proteins, domain coordinates for *SnRK2* sequences from *P. euphratica*, *P. pruinosa* and *A. thaliana* were retrieved using the SMART database (http://smart.embl-heidelberg.de, accessed on 22 December 2024). Based on these coordinates, SnRK2 domain regions were extracted and concatenated into new protein sequences for each species. Multiple sequence alignment was performed using the Clustal W algorithm under default parameters in MEGA-X software (version 11.0.13). A phylogenetic tree was then constructed based on the aligned SnRK2 domain sequences. The resulting tree was visualized and graphically enhanced using the Interactive Tree of Life (iTOL) online tool (https://itol.embl.de/, accessed on 27 December 2024), providing a clear depiction of the evolutionary clustering patterns among SnRK2 proteins from the three species.

### 4.6. Prediction of the Secondary and Three-Dimensional Structures of PeSnRK2 Proteins

Secondary structure prediction of the PeSnRK2 protein family was conducted using the SOPMA tool (https://npsa-prabi.ibcp.fr/cgi-bin/npsa_automat.pl?page=npsa_sopma.html, accessed on 27 December 2024). To further explore the spatial conformation, the three-dimensional (3D) structures of PeSnRK2 proteins were modeled using the SWISS-MODEL server (https://swissmodel.expasy.org/, accessed on 27 December 2024). PeSnRK2 protein was annotated with the kinase domain using PyMOL software (Version 3.1.6.1). These structural predictions provide insights into the folding patterns and potential functional domains of PeSnRK2 proteins.

### 4.7. Transcriptome Sequencing and Data Analysis of PeSnRK2s

Transcriptome sequencing data were obtained from *P. euphratica* seedlings treated with 15% PEG 6000, 100 μmol/L ABA and corresponding control conditions. Quantitative expression data for each treatment were used to extract the expression profiles of *PeSnRK2* gene family members [64]. RNA extraction, cDNA library construction, RNA-seq and primary data analysis were conducted by Frasergen Bioinformatics Co., Ltd. (Wuhan, China). Following library quality assessment, DNA nanoballs were prepared and loaded onto sequencing chips for high-throughput sequencing using the MGI platform. Raw sequencing reads were processed using SOAPnuke software (Version 2.1.0) [65] to remove low-quality data and adapters, yielding high-quality clean reads. Subsequent transcriptomic analysis followed methods described in a previous study [66]. Clean reads were aligned to the *P. euphratica* reference genome using HISAT2 (version 2.1.0) [67]. Gene expression quantification was performed with StringTie (Version 1.3.4d) [68]. Expression levels were normalized and reported as fragments per kilobase of transcript per million mapped reads (FPKM). Differentially expressed genes) were identified using the DESeq2 R package (version 1.30.1). Thresholds were set at |log_2_ Foldchange| > 1 and *p*-value < 0.05. The expression profiles of *PeSnRK2s* were extracted and visualized using TBtools (Version 2.119).

*P. euphratica* seedling cultivation conditions: light cycle, 16 h photoperiod; temperature, 25 °C ± 1 °C; relative humidity, 75%; irrigation, the seedlings were well watered at their roots every 2 weeks for 2 months before treatment. Two-month-old poplar seedlings underwent drought stress treatment with 15% PEG6000 for five days and 100 μM ABA for four hours. Each treatment included a control group with three replicates. After processing is finished, take three leaves of the same size from poplar seedlings and quickly submerge them in liquid nitrogen to freeze. Keep them at –80 °C until they are analyzed. SPARKscript‖RT Plus Kit with gDNA Eraser (SparkJade, Jinan, China) was applied to produce the cDNA through the reverse template of total RNA (1 µg). The reaction mixture (10 μL) contained 5 μL of 2 × SYRB Green qPCR Mix (SparkJade, China), 0.2 μL each of forward and reverse primers, 3.1 μL ddH_2_O and 1.5 μL of cDNA template. Amplification was achieved using the following cycle settings: 95 °C for 30 s, then 45 cycles of 95 °C for 10 s, 55 °C for 10 s, and 72 °C for 30 s for plate reading. qRT-PCR yielded products between 80 bp and 150 bp in size. After normalization with *PeActin*, relative expression was assessed. The 2^−ΔΔCt^ method was applied to data evaluation. The primer sequences used are listed in Table S2 available as Supplementary Data.

### 4.8. Subcellular Localization of PeSnRK2.6

*PeSnRK2.6’s* 2000 bp promoter sequence was cloned into the pGreen0179-35S-MCS-YFP vector. After the ligated vector was transformed into *E. coli* DH5α, a single clone was isolated. The target fragment was then amplified using PCR for positive detection and sequenced to confirm the positive plasmid. The recombinant plasmid 35S::*PeSnRK2.6*-YFP was introduced into *Agrobacterium tumefaciens* strain GV3101 by electroporation. Separately, the strain carrying the nuclear marker plasmid H2B-CFP was cultured overnight in Luria–Bertani (LB) medium supplemented with appropriate antibiotics. The resulting bacterial cultures were inoculated into fresh LB medium containing 50 µM acetosyringone and incubated with shaking until the optical density at 600 nm (OD_600_) reached 1.0–1.2. Bacterial cells were harvested by centrifugation, the supernatant was discarded, and the pellet was resuspended in infiltration buffer (10 mM MES, pH 5.6; 10 mM MgCl_2_·6H_2_O; and 50 µM acetosyringone) to a final OD_600_ of approximately 1.0. The suspension was incubated in darkness at room temperature for 3 h to activate the virulence genes. Equal volumes of *Agrobacterium* cultures harboring 35S::*PeSnRK2.6*-YFP and H2B-CFP were mixed and co-infiltrated into the abaxial surface of 4-week-old *Nicotiana benthamiana* leaves using a 1 mL needleless syringe. Plants were kept in the dark for 12 h post-infiltration and then transferred to standard growth conditions (22 °C, 16 h light/8 h dark) for an additional 36 h. Epidermal leaf tissue was collected and visualized under a laser scanning confocal microscope (TS100, Nikon, Tokyo, Japan). YFP signals were excited with a 488 nm laser, and emission was detected in the 500–550 nm range.

### 4.9. Predicting Protein–Protein Interactions of PeSnRK2.6

Owing to the absence of *P. euphratica* protein entries in the STRING database (http://string-db.org, accessed on 28 December 2024), functional predictions of protein–protein interactions were inferred using homologous proteins from *Arabidopsis thaliana*. The *Arabidopsis* orthologue of PeSnRK2.6 was identified based on sequence similarity, and its corresponding protein sequence was used as a query in the STRING database to construct a putative protein interaction network. The analysis employed default settings, including an interaction confidence score threshold, to visualize predicted interactions with known ABA signaling components and potential regulatory partners [69].

### 4.10. Statistical Analysis

DESeq2 was used for RNA-seq with |log2FC| > 1, *p* < 0.05; 2^−ΔΔCt^ was used for qRT-PCR. Data were represented as means ± standard error. For the analysis of variance (ANOVA) test, * indicates significant differences at based on Sidak’s multiple comparisons test (*p* < 0.05).

## 5. Conclusions

In this study, 11 members of the *PeSnRK2* gene family were identified, with an analysis of their structure, phylogenetic relationships, orthologs, and cis-acting elements. By predicting cis-elements and analyzing the phylogenetic and ortholog relationships of *AtSnRK2* family members in three Salicaceae species, *Arabidopsis thaliana*, and *Oryza sativa*, it was discovered that these structures have been highly conserved throughout evolution. Research indicates that subgroup III members of this family could be crucial in managing responses to abiotic stress through the ABA signaling pathway. The *PeSnRK2.6* gene showed significant upregulation in response to ABA stress, as revealed by both RNA sequencing data and qRT-PCR. Further subcellular localization confirmed *PeSnRK2.6* functions in both nucleus and cytoplasm. This research establishes a foundation for more in-depth investigation of the *PeSnRK2.6* gene in *Populus euphratica* and, by thoroughly analyzing the *PeSnRK2* gene family, enhances our comprehension of abiotic stress responses in this species.

## Figures and Tables

**Table 1:** Characteristics of *PeSnRK2s*.

Gene ID	Number of Amino Acids	Molecular Weight	Theoretical pI	Instability Index	Aliphatic Index	Grand Average of Hydropathicity (GRAVY)	Prediction of Subcellular Localization
*PeuTF05G01370.1*	581	65,986.13	9.57	35.69	82.03	−0.508	Cytoplasm
*PeuTF04G01285.1*	356	40,929.4	5.69	49.81	82.39	−0.496	Cytoskeleton
*PeuTF04G01353.1*	363	41,201.82	4.86	40.24	86.97	−0.325	Cytoplasm
*PeuTF19G00050.1*	361	41,608.17	5.66	50.77	83.99	−0.516	Cytoskeleton
*PeuTF05G01129.1*	362	41,140.95	4.86	40.85	86.13	−0.277	Chloroplast
*PeuTF03G00118.1*	357	41,088.73	6.09	46.02	78.94	−0.527	Cytoskeleton
*PeuTF07G01031.1*	340	38,669.79	5.20	44.09	85.74	−0.370	Cytoplasm
*PeuTF03G00739.1*	338	38,204.71	5.80	37.63	87.07	−0.300	Cytoskeleton
*PeuTF02G00789.1*	412	46,602.21	5.42	40.61	97.21	−0.156	Cytoplasm
*PeuTF09G01080.1*	362	40,992.69	4.76	40.71	89.67	−0.246	Cytoskeleton
*PeuTF04G02065.1*	363	41,612.34	5.61	47.11	84.05	−0.499	Cytoskeleton

## Data Availability

The original contributions presented in this study are included in the article/Supplementary Materials. Further inquiries can be directed to the corresponding author.

## Supplementary Materials

The following supporting information can be downloaded at: https://www.mdpi.com/article/10.3390/ijms262110750/s1.
