# Supplementary material for: Genome-Wide Identification of the SnRK2 Gene Family and Its Response to Abiotic Stress in Populus euphratica
Source: Int J Mol Sci. 2025 Nov 5;26(21):10750. doi: 10.3390/ijms262110750 (PMC12608763; doi:10.3390/ijms262110750)
Supplement: Supplementary file 1 [file ijms-26-10750-s001.zip › ijms-3932221-supplementary.pdf]

Table S1: One-to-one orthologous relationships in *Populus euphratica*

Seq_1	Seq_2	Ka	Ks	Ka/Ks
PeuTF02G00789.1	PeuTF07G01031.1	0.113	1.335	0.085
PeuTF04G01353.1	PeuTF09G01080.1	0.025	0.420	0.059
PeuTF05G01129.1	PeuTF09G01080.1	0.112	1.665	0.067
PeuTF04G01353.1	PeuTF05G01129.1	0.109	1.520	0.072
PeuTF03G00118.1	PeuTF04G02065.1	0.043	0.295	0.147

Table S2: Primer sequences for qRT-PCR

Primer Name	Primer Sequences
PeuTF05G01129.1-F	GATAACCCCAGAGTGTCGCC
PeuTF05G01129.1-R	CATCCATTAGGTCAGCGGGA
PeuTF04G01353.1-F	ACAGTGGGGCCGGGAA
PeuTF04G01353.1-R	TATCCCTCATCAACCTGGCGA
PeuTF09G01080.1-F	ACCCGATCAACCAATGCAAA
PeuTF09G01080.1-R	TCTCCAAGTCCTCCATTTCGTC
PeuTF03G00118.1-F	AAATTCCCCCACCAGTATCGAG
PeuTF03G00118.1-R	AGCCTCTGCATCATCTTCCTT
PeuTF03G00739.1-F	TACTGCACTCGCAACCCAAA
PeuTF03G00739.1-R	GCCATCGTATTCCTTGCGTG
PeuTF05G01370.1-F	GGAGGCAAGCAAACCTGTCT
PeuTF05G01370.1-R	CCGTAGTGGGATTTGCGATG
PeuTF19G00050.1-F	AGATGAGAACGTGGCAAGGG
PeuTF19G00050.1-R	ACCACCTGCAAAAAGATTGAGTT
PeuTF07G01031.1-F	TCGGATTTTTGTAGCTGACCCT
PeuTF07G01031.1-R	GGAAGCTCCTCAAAAACCAGG
PeActin-F	GTCCTCTTCCAGCCATCTC
PeActin-R	TTCGGTCAGCAATACCAGG
